# Galvanic Corrosion of and Ion Release from Various Orthodontic Brackets and Wires in a Fluoride-containing Mouthwash

**DOI:** 10.15171/joddd.2015.030

**Published:** 2015-09-16

**Authors:** Soodeh Tahmasbi, Mohammad Ghorbani, Mahdis Masudrad

**Affiliations:** ^1^Assistant Professor, Department of Orthodontics, Faculty of Dentistry, Shahid Beheshti University of Medical Sciences, Tehran, Iran; ^2^Professor, Department of Materials and Engineering, Sharif University of Technology, Azadi Avenue, Tehran, Iran; ^3^Post-graduate Student, Department of Oral and Maxillofacial Surgery, Ahvaz Jundishapour University of Medical Sciences, Ahvaz, Iran

**Keywords:** Corrosion, orthodontic bracket, sodium fluoride, nickel

## Abstract

***Background and aims.*** This study compared the galvanic corrosion of orthodontic wires and brackets from various manufacturers following exposure to a fluoride mouthwash.

***Materials and methods.*** This study was conducted on 24 lower central incisor 0.022" Roth brackets of four different commercially available brands (Dentaurum, American Orthodontics, ORJ, Shinye). These brackets along with stainless steel (SS) or nickel-titanium (NiTi) orthodontic wires (0.016", round) were immersed in Oral-B mouthwash containing 0.05% sodium fluoride for 28 days. The electric potential (EP) difference of each bracket-wire couple was measured with a Saturated Calomel Reference Electrode (Ag/AgCl saturated with KCl) via a voltmeter. The ions released in the electrolyte weremeasured with an atomic absorption spectrometer. All the specimens were assessed under a stereomicroscope and specimens with corrosion were analyzed with scanning electron microscopy (SEM). Data were analyzed using ANOVA.

***Results.*** The copper ions released from specimens with NiTi wire were greater than those of samples containing SS wire. ORJ brackets released more Cu ions than other samples. The Ni ions released from Shinye brackets were significantly more than those of other specimens (P < 0.05). Corrosion rate of brackets coupled with NiTi wires was higher than that of brackets coupled with SS wires. Light and electron microscopic observations showed greater corrosion of ORJ brackets.

***Conclusion.*** In fluoride mouthwash, Shinye and ORJ brackets exhibited greater corrosion than Dentaurum and American Orthodontics brackets. Stainless steel brackets used with NiTi wires showed greater corrosion and thus caution is recommended when using them.

## Introduction

Wire-bracket systems are commonly used for tooth movements in orthodontic treatments.^1^ These brackets and wires are made of alloys such as stainless steel, chrome-cobalt-nickel and nickel-titanium.^2^ Resistance against corrosion is a must-have quality for orthodontic brackets and wires because corrosion can result in roughness of the appliance, increased friction between the arch wire and slot, discoloration of enamel surface and release of ions from the metal or alloy. Release of ions can lead to the discoloration of adjacent soft tissues, cause local pains or trigger allergic reactions in susceptible subjects. These ions can also cause cytotoxic and biological side effects.^3,4^ These alloys contain nickel which is responsible for the majority of allergic reactions that occur during orthodontic treatments.^2^ It is estimated that 2-2.75% of the population are allergic to nickel and females are more susceptible than males.^5^

Galvanic corrosion is an electrochemical process that occurs when two dissimilar metals come into contact. The metal that has lower resistance against corrosion acts as the anode and dissolves into the electrolyte during an electrochemical reaction and metallic ions are released. In a clinical setting, dissimilar metals and alloys with different EPs like orthodontic brackets and wires or different parts of brackets come into contact in an electrolyte.^3^

In general, corrosion resistance is related to various factors. More importantly, corrosion resistance depends on the manufacturing process, type of alloy and surface characteristics of the appliance. Less importantly, it can be related to the environment where the appliance is going to be used and eventually to its specific application. The type of application of an appliance can exert varying levels of stress and thermal changes to the alloy used.^6-8^It has been shown that release of nickel ions does not depend on the amount of this ion in orthodontic brackets and wires. Rather, it depends on the nature of the alloy and the manufacturing process.^3^Galvanic corrosion is also more dependent on bracket manufacturing rather than bracket composition.^9^On the other hand, the destructive effect of fluoride ion on corrosion resistance of titanium or titanium alloys has been confirmed. Fluoride degrades the protective layer of titanium oxide on the surface of titanium alloys and results in hydrogen absorption leading to degradation of the mechanical properties and reduces corrosion resistance of NiTi wires.^10^ Thus fluoride ion increases the corrosion of brackets and wires and use of fluoride-containing products is common during orthodontic treatments to prevent dental caries.^1,3^

It is necessary to evaluate the behavior of orthodontic brackets and wires when exposed to fluoride mouthwashes.

The present study aimed at evaluating the galvanic corrosion of four different orthodontic brackets (Dentaurum, American Orthodontics, Shinye and ORJ) with SS or NiTi wire in a fluoride mouthwash.

## Materials and Methods

The samples consisted of 24 mandibular central incisor 0.022" Roth brackets of four different manufacturers: Dentaurum (Dentaurum, Ispringen, Germany), American Orthodontics (American Orthodontics, Wisconsin, USA), Shinye (Hangzhou Shinye Orthodontic Products Co., Ltd., Zhejiang, China) and ORJ (Hangzhou ORJ Medical Instruments & Material Co., Zhejiang, China) (n=6). These brackets and 0.016″ stainless steel or NiTi wires (American Orthodontics, Wisconsin, USA) were evaluated in eight groups of three each. The electrolyte was Oral-B fluoride mouthwash (Procter & Gamble, Weighbridge, United Kingdom) with a pH value of 5.6 and 0.05% sodium fluoride. The wire-to-bracket surface area ratio was 1:1 and the excess wire was covered with water-proof nail varnish to prevent electrolyte penetration.

Before weighing, all the brackets and wires were placed in acetone solution for two minutes for surface cleansing. Afterwards, the wires and brackets were separately weighed using Mettler Toledo XS204 scales (Cole-Parmer, IL, USA) with 0.1-mg accuracy and the results were recorded in grams. The percentages of the various metals present in each bracket and wire were measured by a quantometer (ARL, Michigan, USA) with 0.01% accuracy.

Each sample was placed in a separate plate with a Saturated Calomel Reference Electrode (Ag/AgCl saturated with KCl) (Azmiran, Tehran, Iran). Each sample along with its reference electrode was connected to the voltmeter equipped with data logger gathering data every five minutes, using connecting wires. Eighty milliliters of Oral-B fluoride mouthwash was used as the electrolyte in each plate in accordance with ASTM G71-81 (2003) Standard Guide for Conducting and Evaluating Galvanic Corrosion Tests in Electrolytes (ASTM International, USA) and the circuit was completed. The samples were placed in an incubator at 37±0.1°C.

The couple EP difference of each sample with its respective reference electrode was recorded hourly for 28 days. The obtained values were transferred to a computer using a data gathering device (Data Logger, Mv-02, designed by Sharif University of Technology) and saved.

After 28 days, the circuit was opened and the brackets and wires were washed with deionized water with mild pressure for 30 seconds and air-dried. The electrolyte solution was evaluated for the amount of ions released from samples using an Atomic Absorption Spectrometer (GBC Avanta PM, IL, USA).

All the samples were evaluated under a light stereomicroscope (SZH10, Olympus, Tokyo, Japan). The samples which showed corrosion under stereomicroscopic evaluation were also studied with SEM (TESCAN-LMU, Brno, Czech Republic).

For final weighing, the water-proof varnish was wiped off using acetone and the samples were immersed in 10% sulfuric acid solution for two minutes at room temperature followed by another two minutes in sulfuric acid solution at 40˚C. After final irrigation with deionized water for one minute, the samples were air-dried and weighed. In order to determine the corrosion rate, the difference between the primary and final weight of wires and brackets was calculated and the corrosion rate was calculated using the formula below:

Corrosion Rate (mg/dm^2^/day) = ΔW/A/T

where “ΔW” is the weight loss (mg), “A” is the surface area of specimen (dm^2^), and “T” is the exposure time (day).

### Statistical Analysis

ANOVA was used to analyze the differences in mean ion concentrations between the four groups. The proper post hoc test (Tukey HSD, t-test, Tamhane’s test) was applied to assess the differences between the groups.

## Results

### Ion Evaluation

The mean concentration of released ions from different samples is demonstrated in Table 1. The amount of molybdenum ions in all the samples was less than 100 µg/L and the concentration of titanium ions in all the samples was less than 300 µg/L; therefore, these ions were not included in the table.

**Table 1:** The mean concentrations of released ions based on the type of wire and bracket in μg/L

**Wire**	**Bracket**	**Cu**	**Fe**	**Cr**	**N**
**NiTi**	Dentaurum	736.7	53.4	10.0	76.7
	American Ortho	466.7	53.4	10.0	83.4
	Shinye	10.0	416.76	10.0	2133.4
	ORJ	726.7	23.4	10.0	186.7
**Stainless steel**	Dentaurum	16.7	10.0	10.0	10.0
	American ortho	10.0	10.0	10.0	10.0
	Shinye	10.0	253.4	10.0	1866.7
	ORJ	546.7	910.0	206.67	323.4

### Cu Ion 

In general, the concentration of released Cu ions in samples with NiTi wire was greater than that of samples with stainless steel wire. The amount of Cu ions released from ORJ bracket was significantly higher than that of American Orthodontics and Shinye brackets (P=0.032 and P=0.001, respectively).

### Fe Ion

The concentration of Fe ions released from the coupling of NiTi wire and Dentaurum bracket was significantly higher than that of SS wire and Dentaurum bracket coupling (P=0.041). No significant differences were detected in the concentrations of released Fe ions between the coupling of various brackets and SS or NiTi wires (P>0.05).

### Ni Ion 

The concentration of Ni ions released from Shinye brackets was significantly higher than that of the three other brackets (Dentaurum, American Orthodontics and ORJ) (P<0.05). No significant differences were detected in the amounts of Ni ions released between the other three brands.

### Cr Ion

No significant differences were observed in the concentrations of Cr ions between the coupling of various brackets with NiTi or SS wires (P>0.05).

### Evaluation of EP Difference

**Figure 1.**
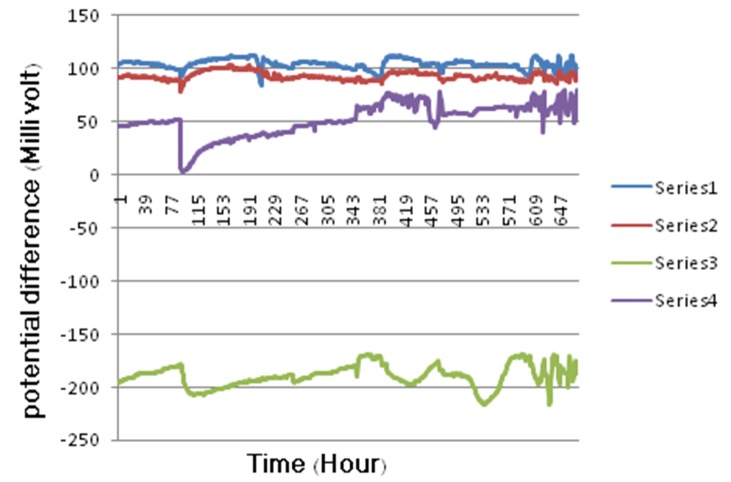
The mean EP differences (in millivolt) ob-tained from the coupling of NiTi wire with different brackets: (1) Dentaurum; (2) American Orthodontics; (3) Shinye; and (4) ORJ brackets.

The differences in EP between samples with Shinye brackets were significantly lower than those of other brackets. In fact, the EP difference for Shinye brackets was negative, in contrast to those of other samples (Figure 1).

### Evaluation of Corrosion Rate

The mean values of corrosion rates are presented in Table 2. The corrosion rate was significantly higher in samples with NiTi wire compared to those with SS wire (P=0.006).

**Table 2:** The mean corrosion rates of brackets and wires based on their manufacturing company in mg/dm^2^/day

**Wire**	**Bracket**	**Corrosion rate of bracket**	**Corrosion rate of wire**
**NiTi**	Dentaurum	4.9	4.32
	American ortho	2.76	5.52
	Shinye	8.04	2.57
	ORJ	7.34	2.14
**Stainless steel**	Dentaurum	1.45	0.29
	American ortho	0.92	0.61
	Shinye	3.21	1.28
	ORJ	3.67	1.53

### Evaluation of Samples under a Light Stereomicroscope 

In one of the samples of NiTi wire and Dentaurum bracket a tarnished area was observed on the wing surface and below the O-ring. Also, the bracket had lost its shine. No changes were observed in the other two samples.

No changes were detected under the light microscope in the three samples of NiTi wires and American Orthodontics brackets and couplings of NiTi wires and Shinye brackets.

In couplings of NiTi wires and ORJ brackets tarnish under the wings and yellow discoloration at the location of O-ring were observed.

**Figure 2.**
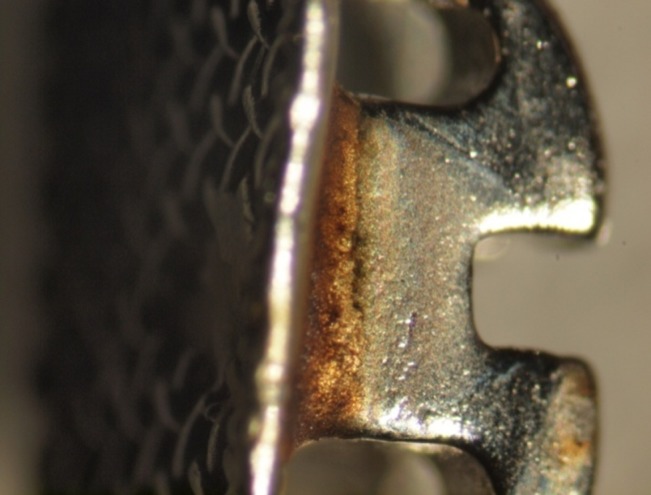
Stereomicroscopic micrograph (×3) of ORJ bracket from group 8 (ORJ bracket & SS wire) after testing.

In samples with SS wires, only the coupling of ORJ brackets and SS wires showed obvious alterations, which were similar to changes observed in the coupling of ORJ brackets with NiTi wires (Figure 2).

### SEM Analysis of Samples

Of all the evaluated samples, 3 brackets and a piece of wire, which showed apparent changes, were selected for SEM analysis as follows:

1. Stainless steel wire of the third sample in group 8
2. ORJ bracket of the third sample in group 8
3. ORJ bracket of the third sample in group 4
4. Dentaurum bracket of the third sample in group 1

### Stainless Steel Wire of the Third Sample in Group 8 (ORJ Bracket & SS Wire)

After testing, surface defects and pitting corrosion were observed on the wire surface.

### ORJ Bracket of the Third Sample in Group 8 (ORJ Bracket & SS Wire)

**Figure 3.**
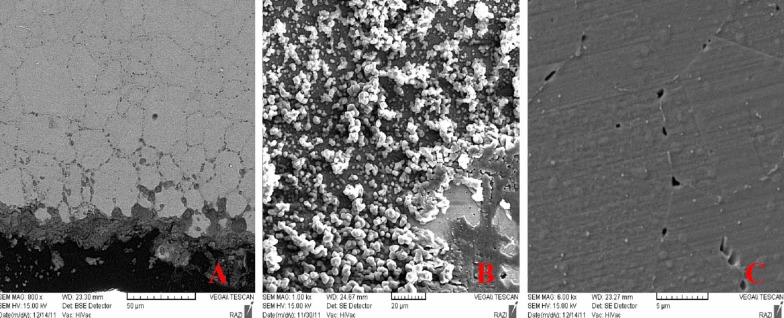
SEM micrograph of ORJ bracket at the location of O-ring from group 8 (ORJ bracket & SS wire): a) after testing and before irrigation (×1000); b) after testing and irrigation (×800); c) after testing and irrigation (×6000).

See Figures 3A,3B and 3C.

Materials observed on the surface of ORJ brackets, after coupling with SS wire and placement in the electrolyte solution, were the products of electrochemical corrosion that appeared yellow under the light microscope. Pitting and intergranular corrosions were also noticed.

### ORJ Bracket of the Third Sample in Group 4 (ORJ Bracket & NiTi Wire)

**Figure 4.**
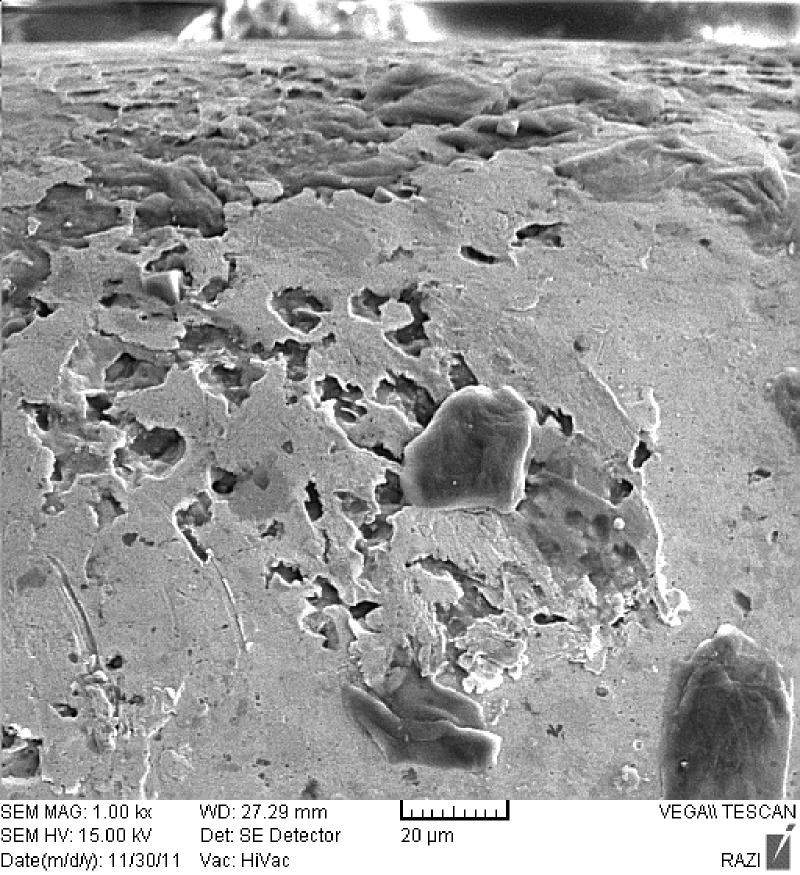
SEM micrograph of ORJ bracket at the location of O-ring from group 4 (ORJ bracket & NiTi wire) after testing and irrigation (×1000).

Some surface defects observed before testing on the brackets were due to their manufacturing process. After testing, pitting corrosion, surface corrosion and numerous defects were observed on the surface of ORJ brackets in the area attached to NiTi wire (Figure 4).

### Dentaurum Bracket of the Third Sample in Group 1 (Dentaurum Bracket & NiTi Wire)

Dentaurum bracket surface became rougher and coarser after the test, compared to its pre-test condition and uniform corrosion was seen in addition to pitting corrosion. The corrosion was greater at the bracket wings compared to other areas.

## Discussion

Various methods are available for evaluation of galvanic corrosion, including immersion tests, potentiostatic and galvanostatic electrochemical tests and also recording of EP differences with reference electrodes given the relation of EP and corrosion rate.^11^In the present study, we used the latter method in addition to the measurement of released ions. The corrosion rate was calculated on the basis of weight loss and the samples were evaluated under a light stereomicroscope and SEM.

In terms of the amount of released ions, the rate of released copper as a result of coupling of brackets with NiTi wires was greater than its released amount when the same brackets were coupled with SS wires. Also, the ORJ brackets released the highest amount of Cu ions.

For Fe ions, a significant difference was detected only when Dentaurum brackets and NiTi wires were placed in the mouthwash. A significantly larger amount of Fe ions were released as the result of this coupling compared to the coupling of Dentaurum and SS wires. Despite the differences in methodology and measuring methods, the mean amount of Fe ions released from the coupling of Dentaurum brackets or American Orthodontics with NiTi wires was similar to the amount reported in a study by Momeni Danaei et al.^12^

The concentration of Ni ions released from Shinye brackets in samples with NiTi or SS wires was greater than the released amount in samples with other brackets. Nickel can cause allergic reactions more than any other metal present in orthodontic appliances. Based on the literature, the concentration of this ion in the oral mucosa of patients with fixed orthodontic appliances is greater than the control group.^5^ Daily intake of nickel from food and drinks is approximately 300-500 µg.^12-14^ On the other hand, it has been confirmed that if the absorption of nickel exceeds 2.5 µg/kg, allergic symptoms will appear.^4^ The amount of Ni ions released in the present study was smaller than the mentioned threshold. This rate was also lower than the concentrated dose required for allergic reactions (600-2500 µg).^15^However, even this small amount of nickel may result in allergic reactions or DNA damage in the oral buccal mucosa cells considering the fact that patients normally use orthodontic appliances for 2 to 3 years and have about 20 brackets in their mouth during this period.^5,12^

In addition, the level of metallic ions released (especially nickel) was greater when SS brackets were coupled with NiTi wires compared to when SS brackets were coupled with SS wires. This is because of the greater EP difference of two dissimilar alloys. In other words, the EP difference between SS and NiTi alloys was greater than that of two different SS alloys. These findings are consistent with the results of Schiff^1^ and Iijima^2^ and indicate that use of SS alloy with NiTi expedites the pace and rate of NiTi alloy corrosion.

In this study, the amount of Cr ions released from wires or brackets was not significantly different between the samples. However, in a study by Barrett and Bishara,^16^it was revealed that SS wires released more Cr ions compared to NiTi wires. This issue has also been discussed in Hwang’s study in 2001.^14^The difference between the results of these studies and ours may be attributed to the different study designs and the solutions used as the electrolyte. EP differences of samples were recorded hourly for 28 days. These rates were significantly lower for couplings of Shinye brackets and SS or NiTi wires and were negative throughout the test. Negative EP difference is indicative of the greater galvanic activity of the mentioned samples and shows that Shinye brackets were more susceptible to corrosion compared to other brackets.

Corrosion rate was higher in brackets coupled with NiTi wire compared to SS wire. This finding further confirms the fact that corrosion of SS brackets is greater when they are coupled with NiTi wires compared to situations where they are coupled with SS wires. The study of Masahiro et al^17^showed that coupling of SUS 304 and NiTi may remarkably accelerate the corrosion of NiTi alloy.

The amount of iron ions released from ORJ samples was greater than the amount released from other brackets. Furthermore, in stereomicroscopic micrographs of these brackets it is observed that the surface of these brackets, especially at the location of the O-ring, has a distinct yellow discoloration which can be attributed to the rusting of these brackets.

Stereomicroscopic and SEM analysis findings demonstrated that the corrosion of Shinye and ORJ brackets was greater than that of Dentaurum and American Orthodontics brackets. The surface alterations observed were also greater in the Shinye and ORJ brackets. Jahanbin et al^4^ reported the greatest corrosion to be at the location of base-wing joint, consistent with our microscopic findings.

On SEM micrographs of Dentaurum brackets, a uniform corrosion was observed on the wing surface of the bracket, whereas on SEM micrographs of ORJ brackets, severe crevice corrosion was observed at the location of O-ring. This type of corrosion occurs when two surfaces are in close contact with each other in low-oxygen conditions, increasing the corrosion on the surface of SS brackets and resulting in salt formation on the surface and release of Fe, Cr and Ni ions from the metal.^3,18,19^Intergranular corrosion is also observed on the surface of these brackets that can lead to staining of SS brackets followed by their weakening and eventual fracture.

## Conclusion

Significant differences were noted in the galvanic corrosion of brackets in a fluoride mouthwash. Regarding nickel release, Shinye brackets and considering microscopic evaluation, ORJ brackets showed greater corrosion than other brackets. Risk and rate of corrosion were greater when SS brackets were coupled with NiTi wires compared to SS wires.

## Acknowledgment

This study was part of a thesis by Mahdis Masudrad for a DDS degree. The thesis supervisor was Dr. Soodeh Tahmasbi and the counseling professor was Dr. Mohammad Ghorbani. This study was approved by the Research Council of Shahid Beheshti University School of Dentistry and funded by the Research Deputy of Shahid Beheshti University of Medical Sciences.
